# Supplementary material for: From Genes to Disease: Reassessing LOXHD1 and AGBL1’s Contribution to Fuchs’ Dystrophy
Source: Int J Mol Sci. 2025 Apr 3;26(7):3343. doi: 10.3390/ijms26073343 (PMC11989410; doi:10.3390/ijms26073343)
Supplement: Supplementary file 1 [file ijms-26-03343-s001.zip › ijms-3516232 Tables S1-S4.pdf]

Supplementary Materials

Table S1 - List of candidate variants in the *LOXHD1* and *AGBL1* genes
Table S2 - List of datasets and samples for RNA-seq analysis
Table S3 - Place of birth of participants in Cohorts 1 and 2
Table S4 - Primer sequences for *AGBL1* and *LOXHD1* SNVs genotyping
Table S5 - Variant validator results

Table S1. List of candidate variants in the *LOXHD1* and *AGBL1* genes. Abbreviations: ND - no data in the corresponding database

Gene	dbSNP ID	HGVS genomic description	Ref allele	Alt allele	Location of the variant in the gene region	Type of aberration	Variant effect	Transcript level description	Protein level description
LOXHD1	rs148468627	NC_000018.10:g.46477695C>T	C	T	exonic	SNV	missense	NM_144612.7:c.6413G>A	NP_653213.6:p.Arg2138Gln
LOXHD1	rs764897088	NC_000018.10:g.46483630G>T	G	T	exonic	SNV	missense	NM_144612.7:c.6112C>A	NP_653213.6:p.His2038Asn
LOXHD1	ND	NC_000018.10:g.46485062C>G	C	G	exonic	SNV	missense	NM_144612.7:c.5953G>C	NP_653213.6:p.Glu1985Gln
LOXHD1	rs201994383	NC_000018.10:g.46507646G>A	G	A	exonic	SNV	missense	NM_144612.7:c.5398C>T	NP_653213.6:p.Arg1800Trp
LOXHD1	rs775871086	NC_000018.10:g.46509757T>A	T	A	exonic	SNV	missense	NM_144612.7:c.5272A>T	NP_653213.6:p.Thr1758Ser
LOXHD1	rs200242497	NC_000018.10:g.46509805C>T	C	T	exonic	SNV	missense	NM_144612.7:c.5224G>A	NP_653213.6:p.Glu1742Lys
LOXHD1	rs372241056	NC_000018.10:g.46521131A>G	A	G	exonic	SNV	missense	NM_144612.7:c.5085+970T>C†	NP_653213.6:p.(?) †
LOXHD1	rs200792636	NC_000018.10:g.46541815G>A	G	A	exonic	SNV	missense	NM_144612.7:c.3874C>T	NP_653213.6:p.Leu1292Phe
LOXHD1	rs564297037	NC_000018.10:g.46566335G>A	G	A	exonic	SNV	missense	NM_144612.7:c.2359C>T	NP_653213.6:p.Arg787Cys
LOXHD1	rs376539851	NC_000018.10:g.46566443G>A	G	A	exonic	SNV	missense	NM_144612.7:c.2251C>T	NP_653213.6:p.Arg751Trp
LOXHD1	rs141932807	NC_000018.10:g.46577732C>T	C	T	exonic	SNV	missense	NM_144612.7:c.1945G>A	NP_653213.6:p.Asp649Asn
LOXHD1	rs540100675	NC_000018.10:g.46579680G>A	G	A	exonic	SNV	missense	NM_144612.7:c.1759C>T	NP_653213.6:p.Arg587Trp
LOXHD1	rs113444922	NC_000018.10:g.46591948G>A	G	A	exonic	SNV	missense	NM_144612.7:c.1639C>T	NP_653213.6:p.Arg547Cys
LOXHD1	rs192376005	NC_000018.10:g.46592017G>A	G	A	exonic	SNV	missense	NM_144612.7:c.1570C>T	NP_653213.6:p.Arg524Cys
LOXHD1	rs566553343	NC_000018.10:g.46639658G>A	G	A	exonic	SNV	missense	NM_144612.7:c.469C>T	NP_653213.6:p.Arg157Cys
LOXHD1	rs2039074525	NC_000018.10:g.46649158C>T	C	T	exonic	SNV	missense	NM_144612.7:c.242G>A	NP_653213.6:p.Ser81Asn
LOXHD1	rs980201296	NC_000018.10:g.46649241A>C	A	C	exonic	SNV	missense	NM_144612.7:c.159T>G	NP_653213.6:p.Asp53Glu
AGBL1	rs185919705	NC_000015.10:g.86674435C>T	C	T	exonic	SNV	nonsense	NM_152336.4:c.3220C>T †	NP_689549.3:p.Arg1074*
AGBL1	rs377248005	NC_000015.10:g.86397372G>A	G	A	exonic	SNV	missense	NM_152336.4:c.2381G>A	NP_689549.3:p.Arg794His †

Gene	dbSNP ID	HGVS genomic description	Ref allele	Alt allele	Location of the variant in the gene region	Type of aberration	Variant effect	Transcript level description	Protein level description
AGBL1	rs181958589	NC_000015.10:g.86674322G>C	G	C	exonic	SNV	missense	NM_152336.4:c.3107G>C †	NP_689549.3:p.Cys1036Ser

† - the variant description differs from that in the original article, it was assigned to the same transcripts and isoforms for each gene: for AGBL1 variants - NM_152336.4 and NP_689549.3, for LOXHD1 - NM_144612.7 and NP_653213.6.

Table S2. List of datasets and samples for RNA-seq analysis

Tissue type	Number of samples	Description	Data set project ID	Samples	Reference
Control endothelium	26	Human corneal endothelium donor samples	PRJNA524323	11 samples	[30]
			PRJNA597343	15 samples	[31]
FECD endothelium	24	Human corneal endothelium patient samples	PRJNA524323	14 samples	[30]
			PRJNA597343	10 samples	[31]
Stem cells	4	Human pluripotent stem cells (hPSCs)	PRJNA512529	SRX5195275	[57]
		Human embryonic stem cells (hESCs)	PRJNA734529	SRR14711147, SRR14711148, SRR14711149	[58]
Neural crest	7	Human neural crest cells (hNCCs) differentiated from H9 hESCs	PRJNA512529	SRX5195276, SRX5195277	[57]
			PRJNA526696	SRR8715391, SRR8715395, SRR8715399	[59]
		hNCCs differentiated from hESCs	PRJNA603497	SRX7642743, SRX7642745	[60]

Table S3. Place of birth of participants in Cohorts 1 and 2

**Place of birth**	**Cohort 1**	**Cohort 2**
Central Federal District	39	102
Volga Federal District	3	12
Siberian Federal District	2	6
Ural Federal District	1	5
Southern Federal District	0	8
Northwestern Federal District	0	8
Far Eastern Federal District	0	9
North Caucasian Federal District	1	1
Ukraine	2	8
Republic of Belarus	0	3
Republic of Uzbekistan	0	3
Turkmenistan	1	0
Republic of Kazakhstan	0	2
Republic of Moldova	1	0
Republic of Lithuania	0	1
Republic of Poland	0	1
Czech Republic	0	1
Republic of Ecuador	0	1
**Sum**	50	171

Table S4. Primer sequences for *AGBL1* and *LOXHD1* SNVs genotyping

dbSNP ID	Primer name	Sequence 5'->3'
rs181958589 and rs185919705	AGBL-f1	ACCACTCAGCTCCCATTATACG
	AGBL-r1	GTCACCAAGCCACATCTTATCA
rs192376005	LOX-f1	TGACCCTGATGAACACTCTGA
	LOX-r1	CTTTCAAGGAGGCTCTGGAGA
rs200242497	LOX1-f2	GTCCATTGACAAGCCAGAGGAACC
	LOX1-r2	CTCTTGGTTCGGGGCCATTTCTAC
